# Predictors of Interstitial Lung Disease in Mixed Connective Tissue Disease

**DOI:** 10.3390/jcm12237481

**Published:** 2023-12-03

**Authors:** Manuel Silvério-António, Joana Martins-Martinho, Ana Teresa Melo, Francisca Guimarães, Eduardo Dourado, Daniela Oliveira, Jorge Lopes, André Saraiva, Ana Gago, Margarida Correia, Ana L. Fernandes, Sara Dinis, Rafaela Teixeira, Susana P. Silva, Carlos Costa, Tiago Beirão, Carolina Furtado, Pedro Abreu, Carmo Afonso, Nikita Khmelinskii

**Affiliations:** 1 Rheumatology Department, Centro Hospitalar Universitário Lisboa Norte, EULAR Center of Excellence, Centro Académico de Medicina de Lisboa, 1649-028 Lisbon, Portugal; manuelqjsa@gmail.com (M.S.-A.); joanamartinsmartinho@gmail.com (J.M.-M.);; 2 Rheumatology Research Unit, Instituto de Medicina Molecular, Faculdade de Medicina, Universidade de Lisboa, Centro Académico de Medicina de Lisboa, 1649-028 Lisbon, Portugal; 3 Rheumatology Department, Unidade Local de Saúde do Alto Minho, 4990-041 Ponte de Lima, Portugal; 4 Rheumatology Department, Centro Hospitalar do Baixo Vouga, 3810-164 Aveiro, Portugal; 5 Egas Moniz Health Alliance, 3810-164 Aveiro, Portugal; 6 Rheumatology Department, Centro Hospitalar Universitário São João, 4200-319 Porto, Portugal; 7 Center for Health Technology and Services Research (CINTESIS), Faculty of Medicine, University of Porto, 4200-450 Porto, Portugal; 8 Rheumatology Department, Hospital Garcia de Orta, 2805-267 Lisboa, Portugal; 9 Rheumatology Department, Centro Hospitalar Universitário de Coimbra, 3004-561 Coimbra, Portugal; 10 Rheumatology Department, Centro Hospitalar Lisboa Ocidental, 1349-019-005 Lisbon, Portugal; 11 Rheumatology Department, Hospital de Braga, 4710-243 Braga, Portugal; 12 Rheumatology Department, Centro Hospitalar Universitário do Algarve, 8000-386 Faro, Portugal; 13 Rheumatology Department, Unidade Local de Saúde da Guarda, 6300-749 Guarda, Portugal; 14 Rheumatology Department, Centro Hospitalar Tondela-Viseu, 3504-509 Viseu, Portugal; 15 Rheumatology Department, Centro Hospitalar Trás-os-Montes e Alto Douro, 5000-508 Vila Real, Portugal; 16 Rheumatology Department, Centro Hospitalar Vila Nova de Gaia/Espinho, 4434-502 Gaia, Portugal; 17 Rheumatology Department, Hospital do Divino Espírito Santo, 9500-370 Ponta Delgada, Portugal; 18 Rheumatology Unit, Unidade Local de Saúde de Castelo Branco, 6000-085 Castelo Branco, Portugal

**Keywords:** interstitial lung disease, mixed connective tissue disease, predictors

## Abstract

Interstitial lung disease (ILD) frequently complicates mixed connective tissue disease (MCTD) and contributes to increased mortality. We aimed to identify predictors of ILD in MCTD patients. This is a nationwide, multicentre, retrospective study including patients with an adult-onset MCTD clinical diagnosis who met Sharp’s, Kasukawa, Alarcón-Segovia, or Kahn’s diagnostic criteria and had available chest high-resolution computed tomography (HRCT) data. Univariate and multivariate analyses were conducted. We included 57 MCTD patients, with 27 (47.4%) having ILD. Among ILD patients, 48.1% were asymptomatic, 80.0% exhibited a restrictive pattern on pulmonary function tests, and 81.5% had nonspecific interstitial pneumonia on chest HRCT. Gastroesophageal involvement (40.7% vs. 16.7%, *p* = 0.043) and lymphadenopathy at disease onset (22.2% vs. 3.3%, *p* = 0.045) were associated with ILD. Binary logistic regression identified lymphadenopathy at disease onset (OR 19.65, 95% CI: 1.91–201.75, *p* = 0.012) and older age at diagnosis (OR 1.06/year, 95% CI: 1.00–1.12, *p* = 0.046) as independent ILD predictors, regardless of gender and gastroesophageal involvement. This study is the first to assess a Portuguese MCTD cohort. As previously reported, it confirmed the link between gastroesophageal involvement and ILD in MCTD patients. Additionally, it established that lymphadenopathy at disease onset and older age at diagnosis independently predict ILD in MCTD patients.

## 1. Introduction

Mixed connective tissue disease (MCTD) is a rare condition characterized by elevated levels of autoantibodies targeting the U1-ribonucleoprotein complex (U1-RNP). This complex connective tissue disease shares overlapping clinical features with systemic sclerosis (SSc), systemic lupus erythematosus (SLE), and polymyositis [1]. Patients with MCTD often present with a combination of symptoms, including arthritis, myositis, Raynaud’s phenomenon, puffy fingers, oesophageal hypomotility or dilation, and interstitial lung disease (ILD) [2]. As a major complication of MCTD, ILD impacts as many as 78% of patients and is linked to increased mortality rates in comparison to MCTD patients who do not experience ILD [2,3,4,5,6]. Identifying potential predictors of ILD is crucial to detect which MCTD patients are most at risk. Previous studies that investigated ILD clinical associations and predictors reported that older MCTD patients and those presenting Raynaud’s phenomenon, dysphagia, elevated C-reactive protein (CRP), anti-Ro52 autoantibodies, and giant capillaries in nailfold capillaroscopy had a higher prevalence of ILD [5,6,7,8,9,10]. In this study, we further expand this line of research in a nationwide Portuguese cohort of MCTD patients. In particular, we aimed to identify clinical or serological predictors of ILD in MCTD patients, thus contributing to the early detection of ILD and mitigation of its outcomes.

## 2. Materials and Methods

### 2.1. Study Population

This multicentre study was conducted across 16 Portuguese Rheumatology departments. The inclusion criteria were as follows: (i) patients diagnosed with MCTD who were ≥18 years old at the time of diagnosis; (ii) fulfillment of at least one of the four diagnostic criteria sets for MCTD: Sharp’s, Kasukawa, Alarcón-Segovia, or Kahn’s; and (iii) available chest high-resolution computed tomography (HRCT) data. There were no exclusion criteria.

### 2.2. Data Collection

Retrospective data were collected from clinical files. Clinical features were recorded as those present at the time of diagnosis (“onset-<manifestation>”) and those occurring at any time throughout the disease course (“<manifestation>”). Gastroesophageal involvement encompassed dysphagia, gastroesophageal reflux, or dysmotility confirmed through manometry. Lymphadenopathy was considered present if detected in any site during physical examination or confirmed through imaging findings, after exclusion of alternative diagnoses. Muscle involvement was considered if the patient presented proximal muscle weakness or elevated creatine kinase levels, or if myopathic changes were observed on electromyography or muscular biopsy. Renal involvement was identified by urinary sediment alterations, membranous glomerulonephritis, proliferative glomerulonephritis, or tubulointerstitial nephritis.

Patients were classified as having ILD upon confirmation through chest HRCT. Chest HRCT data were categorized based on with or without the evidence of ILD. For patients with ILD, data regarding the predominant HRCT pattern at the time of ILD diagnosis, including nonspecific interstitial pneumonia (NSIP), usual interstitial pneumonia (UIP), and lymphocytic interstitial pneumonia (LIP), were recorded. For patients without ILD, the last available chest HRCT was recorded. The data of pulmonary function tests (PFTs) were categorized as either normal, presenting a restrictive (total lung capacity [TLC] < 80%, forced vital capacity [FVC] < 80%, and forced expiratory volume in the first second [FEV1]/FVC > 70% of the predicted value), obstructive (TLC > 80%, FVC > 80%, and FEV1/FVC < 70% of the predicted value), or a mixed pattern (FVC < 80% and FEV1/FVC < 70% of the predicted value). Recorded PFTs corresponded to the worst available results throughout the disease course.

### 2.3. Statistical Analysis

Categorical variables are presented as absolute numbers/percentages, whereas continuous data are presented as mean (standard deviation) or median (interquartile range) for variables exhibiting skewed distribution. The Shapiro–Wilk test was used to test normal distribution for continuous data. In the univariate analysis, comparisons between categorical variables were conducted using Chi-square or Fisher exact tests, while comparisons between categorical and continuous variables, whether normally distributed or not, were assessed using Student’s *t*-test or Mann–Whitney U test, respectively. Multivariate analysis was carried out via binary logistic regression modelling. The linearity of the continuous variables concerning the logit of the dependent variable was assessed via the Box-Tidwell procedure. Cases with missing information and outliers were excluded from the multivariate analysis to meet the necessary assumptions for regression validity. The statistical analysis was executed using SPSS version 25, with statistical significance defined as a 2-sided *p*-value of less than 0.05.

### 2.4. Ethical Approval

This study was approved by the Ethics Committee of all participant centres and conducted in accordance with the principles of the Declaration of Helsinki. Written informed consent was waived by the Ethics Committee, considering the retrospective nature of the study and the appropriate measures that were taken to ensure compliance with the General Data Protection Regulation (GDPR) of the European Union (EU). Patient confidentiality was maintained through the pseudonymization of data, and access to the full clinical files was limited to the patient’s attending physician, minimizing any risk of breaching confidentiality or impacting the welfare and rights of the patients in accordance with GDPR guidelines.

## 3. Results

Fifty-seven patients were enrolled in the study, 84.2% of whom were female, and 64.9% were of Caucasian descent. The median (IQR) age at the time of MCTD diagnosis was 36.3 (17.4) years old, and the median (IQR) disease duration up to the point of data collection was 4.8 (7.7) years. The majority of patients (91.2%) fulfilled the Kasukawa diagnostic criteria, while 38.6% met the Alarcón-Segovia, 29.8% met the Kahn, and 28.1% met the Sharp’s diagnostic criteria. The most common symptoms at the time of MCDT diagnosis and throughout the disease course were, respectively, arthralgia in 89.5% and 94.7% of patients, arthritis in 62.5% and 73.8%, puffy finger in 57.9% and 61.4%, sclerodactyly in 35.7% and 45.6%, myositis in 29.8% and 43.9%, and Raynaud’s phenomenon in 19.3% and 98.2% patients.

ILD was reported in 27/57 (47.4%) of patients. In patients with ILD, 74.1% were female, and 72.0% were of Caucasian descent. The median (IQR) age at the time of MCDT diagnosis was 39.7 (27.4) years old, and the median (IQR) disease duration up to the point of data collection was 4.6 (6.8) years. The median (IQR) disease duration until ILD diagnosis was 1.0 (4.3) years. Of these patients, 18.2% were either active or ex-smokers (Table 1). There were no significant differences regarding sex, race, or disease duration between patients with and without ILD. Among the patients with ILD, 13/27 (48.1%) were asymptomatic. In the symptomatic group (14/27), dyspnoea affected 13/14 (92.9%) patients, cough affected 7/14 (50.0%), and pleuritic chest pain affected 1/14 (7.1%). PFTs were reported in 25/27 (92.6%) MCDT patients with ILD, with 20/25 (80.0%) exhibiting a restrictive pattern. Normal PFTs were reported in 3/25 (12.0%) patients, and an obstructive pattern was detected in 2/25 (8.0%) patients. The most common chest CT pattern was NSIP in 22/27 (81.5%) patients, followed by UIP in 4/27 (14.8%) patients, and LIP in 1/27 (3.7%) patients.

Among the patients without ILD (30/57; 52.6%), only 3/30 (10%) reported respiratory symptoms—cough in two (6.7%) patients and pleuritic chest pain in one (1.3%) patient. PFTs were reported in 23/30 (76.7%) MCDT patients without ILD, with 14/23 (60.9%) having normal PFTs. A restrictive pattern was reported in 6/23 (26.1%) patients, an obstructive pattern in 2/23 (8.7%) patients, and a mixed pattern in 1/23 (4.3%) patients. All but one patient with reported atelectasis had normal chest HRCT. 

In univariate analysis, gastroesophageal involvement (40.7% vs. 16.7%, *p* = 0.043) and onset-lymphadenopathy (22.2% vs. 3.3%, *p* = 0.045) were significantly associated with ILD (Table 1). Additionally, a non-significant tendency for anti-citrullinated protein antibodies (ACPA) positivity in patients with ILD (17.4% vs. 0%, *p* = 0.05) was present.

The binary logistic regression model that predicted ILD included 56 patients. The model explained 36.5% (Nagelkerke R2) of the variance in ILD and correctly classified 75% of all cases. The identified independent predictors of ILD were onset-lymphadenopathy (OR 19.65, 95% CI: 1.91–201.75, *p* = 0.012) and older age at diagnosis (OR 1.06/year, 95% CI: 1.00–1.12, *p* = 0.046), regardless of sex and gastroesophageal involvement (Table 2). 

## 4. Discussion

The identification of predictors of ILD in MCTD patients is still poorly explored in the literature. The present study aimed to address this gap by examining clinical and serologic predictors of ILD in a nationwide cohort of MCTD patients. Our main findings were that lymphadenopathy at disease onset and older age at diagnosis are independent predictors of ILD in MCTD patients. Also, we confirmed the previously reported association between gastroesophageal involvement and ILD.

Almost half of the MCTD patients in the cohort had ILD, which is consistent with previous reports [2,3,4,5]. Around half of the documented ILD patients were asymptomatic, which was slightly higher than in other studies [3,11]. Concerning the imaging findings, NSIP was the most frequently found imaging pattern in our cohort, which is consistent with previous reports that identified reticular and ground-glass opacities as the most prevalent changes in chest CT [3,12,13]. In addition, our study identified chest HRCT changes suggestive of UIP (honeycombing pattern and traction bronchiectasis) in the range of what has been previously reported by other studies (14.8% vs. 13–58%) [2,12].

Two independent predictors of ILD in MCTD patients were identified: onset-lymphadenopathy and older age at diagnosis. Previous studies have associated a higher prevalence of ILD in MCTD patients with older age at diagnosis [2,6,14]. Late diagnosis of MCTD can result in the progression of the disease, leading to the involvement of more organs, including the lungs. Additionally, this study is the first to identify onset-lymphadenopathy as a predictor of ILD. The presence of lymphadenopathy at disease onset may reflect a highly systemic inflammatory condition. Also, although it did not reach statistical significance, we observed higher erythrocyte sedimentation rate and gamma globulin levels in patients with ILD. An association between a highly inflammatory state, demonstrated by elevated CRP, and ILD was previously described, suggesting that ILD may be more common in patients with higher levels of systemic inflammation [6].

Our study also revealed an association between ILD and gastroesophageal involvement, which has been extensively reported [5,6,7,12,14]. In contrast to previous studies, our results did not show an association between ILD and Raynaud’s phenomenon, anti-Ro52 antibodies, and scleroderma pattern on nailfold capillaroscopy [5,6,7,8,9,10]. Different study inclusion criteria may explain these differences. Most of the previous reports limited the study population to patients fulfilling the Kasukawa criteria [5,7,10]. Celińska-Löwenhoff et al. also included all four diagnostic criteria for MCTD but, unlike our study, excluded those patients fulfilling other connective tissue disease classification criteria [9]. Although our study did not find any serologic predictor for ILD, there was a non-significant tendency for ACPA positivity in MCTD patients with ILD, akin to rheumatoid arthritis [15].

This study represents the inaugural evaluation of a Portuguese MCTD cohort, contributing to the existing literature on ILD among MCTD patients. We identified two independent predictors of ILD in MCTD patients, namely onset-lymphadenopathy and older age at diagnosis, and confirmed an association between gastroesophageal involvement and ILD. Thus, clinicians should consider ILD screening regardless of symptoms, particularly in older patients at diagnosis and patients presenting with lymphadenopathy at disease onset. The small sample size and the retrospective design are significant limitations of the present study. Due to the lack of standardized screening recommendations for ILD in MCTD patients, an underreporting of MCTD patients without ILD or with mild and asymptomatic disease cannot be excluded. This could have impacted the population’s prevalence of ILD and overall characterization. The absence of predetermined time points for data collection, although allowing a comprehensive overview of the patients’ history, introduces a limitation and hinders the longitudinal evaluation of PFTs and HRCT in this MCTD cohort. The possibility that, in some cases, both lymphadenopathy and ILD may have been identified in a single exam cannot be excluded, thereby constraining its predictive efficacy. Nevertheless, the associations found in this study remain significant and warrant further investigation in a larger cohort with a prospective design.

## Figures and Tables

**Table 1:** Characterization of the MCTD patients with and without ILD.

	Without ILD N = 30	With ILD N = 27	*p*-Value
Sociodemographic characteristics, n/N			
African ancestry	9/28	7/25	0.743
Age at diagnosis, median (IQR), years	34.8 (15.0)	39.7 (27.4)	0.099
Disease duration ^a^, median (IQR), years	4.9 (10.2)	4.6 (6.8)	0.371
Female sex	28/30	20/27	0.070
Smoking status, n/N			
Active or ex-smoker	2/26	4/22	0.392
Clinical manifestations, n/N			
Arthralgia Onset Ever	26/30 29/30	25/27 25/27	0.673 0.599
Arthritis Onset Ever	19/29 24/30	16/27 18/27	0.629 0.254
Chronic disease anaemia ^b^ Onset Ever	7/29 9/29	9/27 11/27	0.447 0.449
Cutaneous thickening Onset Ever	7/29 11/30	5/27 7/27	0.609 0.384
Digital ulcers Onset Ever	6/30 6/30	5/27 9/27	0.887 0.254
Erosions Onset Ever	1/24 1/25	0/23 1/23	1.000 1.000
Fever ^c^ Onset Ever	3/30 5/30	7/27 8/27	0.167 0.244
Gastroesophageal involvement ^d^ Onset Ever	1/30 5/30	3/27 11/27	0.336 **0.043**
Leukopenia ^e^ Onset Ever	8/30 13/30	7/27 11/27	0.949 0.843
Lymphadenopathy ^f^ Onset Ever	1/30 6/30	6/27 8/27	**0.045** 0.399
Muscle involvement ^g^ Onset Ever	8/30 12/30	9/27 13/27	0.583 0.536
Neuropathy Onset Ever	2/30 2/30	1/27 3/27	1.000 0.660
Puffy fingers Onset Ever	17/30 18/30	16/27 17/27	0.843 1.000
Pulmonary hypertension ^h^ Onset Ever	0/30 2/29	1/27 3/27	0.474 0.664
Raynaud phenomenon Onset Ever	28/30 29/30	25/27 27/27	1.000 1.000
Renal involvement ^i^ Onset Ever	1/30 3/30	1/27 3/27	1.000 1.000
Sicca syndrome Onset Ever	5/30 10/30	8/27 8/27	0.244 0.764
Sclerodactyly Onset Ever	11/29 14/30	9/27 12/27	0.720 0.866
Thrombocytopenia ^j^ Onset Ever	2/29 5/30	0/27 2/27	0.492 0.427
Weight loss ^k^ Onset Ever	5/30 6/30	10/27 11/27	0.081 0.087
Serogical characteristics, n/N			
Anti-beta-2 glycoprotein 1	4/28	0/26	0.112
Anti-cardiolipin	3/28	0/26	0.237
Anti-citrullinated protein antibodies	0/24	4/23	0.050
Anti-dsDNA	10/30	5/27	0.205
Anti-La	13/30	10/27	0.629
Anti-Ro	2/30	3/27	0.660
Anti-Sm	8/28	6/27	0.589
Lupus anticoagulant	3/27	1/26	0.610
Rheumatoid factor	12/29	14/27	0.432
Onset erythrocyte sedimentation rate in mm/hr, median (IQR) ^l^	60.5 (70.3)	80 (73)	0.694
Onset gamma globulin in g/dL, median (IQR) ^l^	1.8 (1.1)	2.5 (1.1)	0.456
Capillaroscopic pattern, n/N			
Scleroderma pattern	7/23	8/15	0.190

^a^ Disease duration up to the point of data collection; ^b^ chronic disease anaemia if haemoglobin < 12 mg/dL, after exclusion of alternative diagnoses; ^c^ fever if axillary temperature > 38 °C or tympanic temperature > 38.3 °C; ^d^ gastroesophageal involvement included dysphagia, gastroesophageal reflux, or dysmotility confirmed by manometry; ^e^ leukopenia if leukocyte count < 4000/uL; ^f^ lymphadenopathy at any site detected upon physical examination or based on imaging finding, after exclusion of alternative diagnoses; ^g^ muscle involvement included proximal muscle weakness, creatine kinase elevation, or myopathic changes in electromyography or muscular biopsy; ^h^ all patients reported intermediate to high echocardiographic probability for pulmonary hypertension; ^i^ renal involvement included urinary sediment alterations, membranous glomerulonephritis, proliferative glomerulonephritis, or tubulointerstitial nephritis; ^j^ thrombocytopenia if platelet count < 150,000/uL; ^k^ unintentional weight loss of at least 5% within the preceding 6 months. ^l^ Data regarding onset erythrocyte sedimentation rate and gamma globulin levels reported in 8/30 patients without ILD and 8/27 patients with ILD. Values in bold indicate statistical significance at the 0.05 level (*p* < 0.05).

**Table 2:** Multivariate analysis according to interstitial lung disease involvement.

Multivariate Analysis	95% CI	OR	*p*-Value
Independent predictors of interstitial lung disease			
Age at diagnosis	1.00–1.12	1.06	**0.046**
Gastroesophageal involvement	0.56–9.86	2.35	0.243
Male sex	0.45–17.25	2.80	0.268
Onset-lymphadenopathy	1.91–201.75	19.65	**0.012**

Values in bold indicate statistical significance at the 0.05 level (*p* < 0.05).

## Data Availability

The data underlying this article will be shared upon reasonable request to the corresponding author.
